# Patient perspectives on home-based rehabilitation exercise and general physical activity after total hip arthroplasty: A qualitative study (PHETHAS-2)

**DOI:** 10.12688/f1000research.51684.4

**Published:** 2022-08-04

**Authors:** Anne Grøndahl Poulsen, Janni Dahlgaard Gravesen, Merete Nørgaard Madsen, Lone Ramer Mikkelsen, Thomas Bandholm, Camilla Blach Rossen

**Affiliations:** 1 Research Unit, Elective Surgery Center, Silkeborg Regional Hospital, Silkeborg, 8600, Denmark; 2 Department of Clinical Medicine, Aarhus University, Aarhus, 8000, Denmark; 3 Physical Medicine & Rehabilitation Research – Copenhagen (PMR-C), Department of Physical and Occupational Therapy, Clinical Research Center and Department of Orthopedic Surgery, Copenhagen University Hospital, Amager and Hvidovre, Hvidovre, 2650, Denmark

**Keywords:** total hip arthroplasty, rehabilitation, qualitative research, patient perspective

## Abstract

**Background: **Home-based rehabilitation exercise following Total Hip Arthroplasty (THA) shows similar outcomes compared to supervised outpatient rehabilitation exercise. Little is known about patients' experiences with home-based rehabilitation, and this study aimed to investigate how patients perceived home-based rehabilitation exercise and general physical activity after THA, focusing on facilitators and barriers.

**Methods:** Semi-structured interviews of qualitative design were conducted with 22 patients who had undergone THA and who had performed home-based rehabilitation exercise. The study took place in a regional hospital in Denmark between January 2018 and May 2019. Data were analyzed using an interpretive thematic analysis approach, with theoretical underpinning from the concept ‘conduct of everyday life’. The study is embedded within the Pragmatic Home-Based Exercise Therapy after Total Hip Arthroplasty-Silkeborg trial (PHETHAS-1).

**Results:** The main theme, ‘wishing to return to the well-known everyday life’, and four subthemes were identified. Generally, participants found the home-based rehabilitation exercise boring but were motivated by the goal of returning to their well-known everyday life and performing their usual general physical activities, though some lacked contact to physiotherapist. Participants enrolled in the PHETHAS-1 study used the enrollment as part of their motivation for doing the exercises.

Both pain and the absence of pain were identified as barriers for doing home-based rehabilitation exercise. Pain could cause insecurity about possible medical complications, while the absence of pain could lead to the rehabilitation exercise being perceived as pointless.

**Conclusions: **The overall goal of returning to the well-known everyday life served as a facilitator for undertaking home-based rehabilitation exercise after THA along with the flexibility regarding time and place for performing exercises. Boring exercises as well as both pain and no pain were identified as barriers to the performance of home-based rehabilitation exercise. Participants were motivated towards performing general physical activities which were part of their everyday life.

## Introduction

Total hip arthroplasty (THA) is a common surgical intervention in Western countries. It is often performed as fast-track surgery and the number of THAs has been rising. ^1^^,^^2^ Fast-track surgery has proven to be effective in terms of reducing costs, length of hospital stay, morbidity, and convalescence. ^3^^,^^4^ In Denmark alone, 11,000 THAs are performed every year, ^5^ with patients routinely being discharged from the hospital within two days of surgery. ^6^

Rehabilitation exercise is a customary part of the postoperative program for patients undergoing THA, in the expectation that it will reduce pain and increase mobility. ^7^ This is also the case in Denmark, with each hospital having different procedures. Some hospitals refer patients to supervised rehabilitation exercise in the municipality while others recommend home-based rehabilitation exercise after initial instruction is provided. ^8^ Level 1a-evidence from systematic reviews show that supervised exercise after THA provides no additional benefit compared to home-based rehabilitation exercise after initial instruction, when considering patient-reported function, pain, health-related quality of life, or performance-based functions. ^9^^,^^10^ Additionally, home-based rehabilitation is presumably less expensive than supervised rehabilitation, and with rising healthcare costs, one might expect home-based rehabilitation exercise to become even more prevalent in the future.

There are indications that adherence to home-based rehabilitation exercise is low which might affect outcome. Jan *et al.* found that only half of their included participants performed 50% or more of the prescribed home-based rehabilitation exercise. ^11^ They also found that the high compliance group showed greater improvements in muscle strength and functional ability compared to the low compliance group. ^11^ Studies also find that patients have low expectations to obtain greater levels of activity than pre-operatively, ^12^ yet we know little about patients' perspectives, including facilitators and barriers, regarding home-based rehabilitation exercise and general physical activity after THA.

The PHETHAS studies were founded to support and optimize clinical pathways with patients rehabilitating at home after THA. PHETHAS-1 ( ClinicalTrials.gov NCT03109821, April 12, 2017) quantitatively investigates the physical outcomes of performing a home-based rehabilitation exercise program, which after initial instruction was performed without further supervision, ^12^ while this study, PHETHAS-2, qualitatively investigates how patients perceive this home-based rehabilitation exercise and general physical activity after THA, focusing on facilitators and barriers.

## Methods

### Ethics statement

The study complies with the declaration of Helsinki ^14^ and was approved by The Ethics Committee of Central Denmark Region and the Danish Data Protection Agency (ref. no: 1-16-02-589-15). The interviewer obtained written informed consent from participants prior to the interviews being conducted. Consent included participation in the interview, and consent for the participant’s data being used in analysis, including publication of anonymized quotations.

### Theoretical underpinning

The concept ‘conduct of everyday life’ from critical psychology ^15^^,^^16^ was chosen as the theoretical underpinning for this study. ‘Conduct of everyday life’ is an overall concept that embraces the complexity of an individual's everyday life across contexts. ^15^^,^^16^ It includes the different aspects of a person's everyday life, which could be working, performing general physical activities, or home-based rehabilitation exercises. According to theory, the individual person will prioritize activities based on what he/she considers will contribute to their subjective understanding of ‘quality of life’. ^15^^,^^16^

Using ‘conduct of everyday life’ as the theoretical underpinning provides the potential to elucidate how patients integrate both general physical activity and home-based rehabilitation exercise into their everyday lives in the rehabilitation period, thereby informing on possible patient perceived barriers and facilitators for performing the rehabilitation exercise.

We defined home-based rehabilitation exercise as a plan of physical activities in which patients received an initial instruction at the hospital and was performed at the participants’ home without further supervision. It is designed and prescribed to meet specific therapeutic goals. Its purpose is to restore normal musculoskeletal function or to reduce pain caused by diseases or injuries. This definition is synonymous with the Medical Subject Headings (MeSH) term ‘Exercise therapy’ as defined in the PubMed MeSH database. ^17^ Our definition is also in alignment with the World Health Organization’s description of ‘exercises’ as a subcategory of ‘physical activity’. ^18^ In this study we distinguish this type of prescribed rehabilitation exercise from general physical activity undertaken while working, playing, gardening, and engaging in leisure time activities.

### Participants and recruitment

Participants were recruited from a Danish Regional Hospital in the period January 2018 to September 2019. In terms of study inclusion and exclusion criterion, adults > 18 years who had undergone a primary THA due to osteoarthritis were included, but any patients who had been referred for supervised rehabilitation were excluded. The participants also needed to understand written and spoken Danish. The participants were purposely sampled ^19^ with the aim of reflecting the gender and ages of typical THA patients ^20^ as well as representation of both patients from standard care and patients participating in Phethas-1 (see below). Furthermore, sampling was based on the ongoing analysis including themes that were under continual development. When thick descriptions of the themes were obtained and no new themes were identified, recruitment was terminated. ^24^ All invited patients accepted participation.

**Figure 1.**
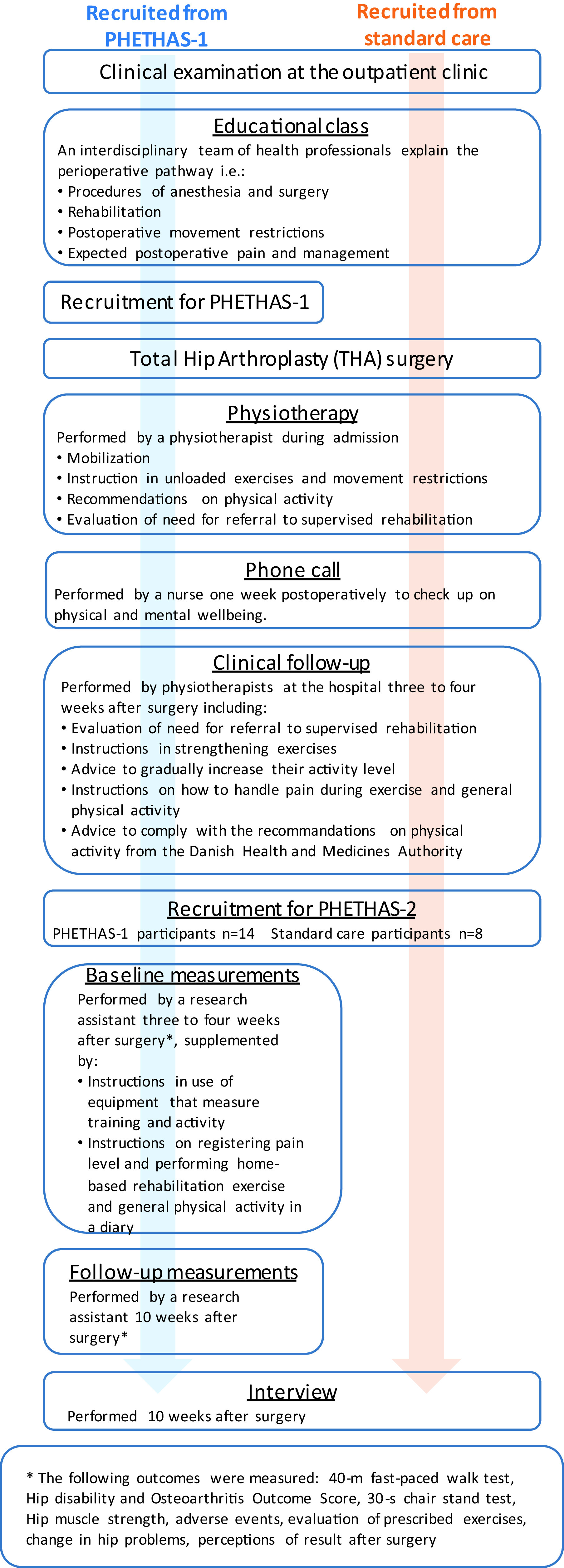
Flowchart of the study design in Pragmatic Home-Based Exercise Therapy after Total Hip Arthroplasty – Silkeborg (PHETHAS-2).

As this study was embedded in the PHETHAS-1 study, participants were recruited from patients enrolled in PHETHAS-1 by the researcher responsible for PHETHAS-1 (MNM) in a face-to-face approach. Participants in PHETHAS-1 may have been more motivated to exercise than the average THA patient and hence may have been more adherent than those who decline participation in clinical exercise trials. Furthermore, it could be speculated that outcome assessments in PHETHAS-1 (exercise dairy, elastic band sensor and physical testing) may increase adherence for participants compared to standard care patients, who did not have a sensor on their elastic band, an exercise diary, nor were they physically tested. With this in mind, and to avoid gathering data from participants of PHETHAS-1 only, we recruited an additional eight participants from standard care. Standard care participants were recruited in a face-to-face approach by physiotherapists responsible for the standard care pathway at the hospital (see Figure 1). A total of 22 participants were included. No participants dropped out. All participants were instructed to perform the exact same home-based rehabilitation exercise. This included an instruction before hospital discharge, where the patients were instructed to perform daily unloaded hip exercises for the first three weeks. At the clinical follow up after three weeks, the patients were instructed to perform strengthening exercises every second day, supplemented with daily balance exercises and stretching of the hip flexor muscles. The strengthening exercises were hip abduction, hip flexion and hip extension with elastic band resistance plus sit-to-stand exercise. Details of this home-based rehabilitation exercise have previously been published, ^13^ see Figure 1 for an overview.

### Data collection

The interviews took place during the period February 2018 to December 2019. Demographic data in terms of age, gender and working status (retired or not) were collected. The demographics of the participants are illustrated in Table 1.

**Table 1.** Characteristics of study participants.

	Participants recruited from standard care (n = 8)	Participants recruited from PHETHAS-1 ^*^ (n = 14)	All participants (n = 22)
Gender (female/male) Number (percent)	6/2 (75/25)	4/10 (29/71)	10/12 (45/55)
Mean age (years) Median (range)	70 (42-82)	69 (48-80)	69 (42-82)
Retired (yes/no) Number (percent)	5/3 (63/37)	10/4 (71/29)	15/7 (68/32)

* Pragmatic Home-Based Exercise after Total Hip Arthroplasty – Silkeborg (PHETHAS-1) is a trial investigating the preliminary efficacy of home-based rehabilitation using elastic band exercise on performance-based function after Total Hip Arthroplasty.

Individual interviews with the participants were conducted to gain an in-depth knowledge of their experiences with home-based rehabilitation exercise and general physical activity after THA. ^21^ The interviews were guided by a semi-structured interview guide, ^21^^,^^22^ which is provided in *Extended data*. ^23^ The interview guide was informed by existing knowledge in the field of THA along with the theoretical concept ‘conduct of everyday life'. The first interviews conducted were planned for pilot testing the interview guide, but since no changes were found necessary regarding interview guide and procedure, these interviews were included for analysis.

Data collection and recruitment was guided by a concurrent data analysis. ^24^ The interviews were conducted 10 weeks postoperatively. At this point in time patients had experience performing the home-based exercise program and 10 weeks postoperatively was also the point of time for the physical testing of PHETHAS-1 participants and thereby the participatory burden for these participants was minimized.

Participants enrolled in PHETHAS-1 were physically tested at the hospital 10 weeks postoperatively and were individually interviewed afterwards in a private meeting room. Participants following standard care were interviewed in their homes ( Figure 1). Occasionally a spouse was present when the interview was conducted, but they did not interfere or participate in the interview. Interviews were audio recorded and lasted an average of 43 minutes (20–67 minutes). The interviews were conducted by AG, JG and CR, who are all female researchers. AG is a physiotherapist with experience in rehabilitation following THA and both JG and CR are registered nurses without specific experience with rehabilitation following THA. AG and JG, both research assistants holding a master’s degree, are trained in the qualitative field of research and are trained interviewers. They were supervised in between interviews by CR, who is a researcher holding a PhD, and an experienced qualitative researcher and interviewer.

### Data analysis

Data were thematically analyzed. This is a method for identifying and analyzing patterns of meaning across data. ^25^^,^^26^ The audio recordings were transcribed verbatim by assistants. The transcripts were subsequently read and corrected by AG and JG, while listening back on the recordings. Initially, the interviews were manually coded by AG and JG, with supervision and input from CR. The coding process was carried out both deductively and inductively. The deductive part of the analysis was guided by theory e.g. how the participants integrate home-based rehabilitation exercise and general physical activity as part of their everyday life during the rehabilitation period. This included which activities they prioritized and why, along with factors serving as motivators or barriers respectively. The inductive part of analysis added an openness for other themes of importance in the dataset. ^25^^,^^26^ The coding process generated potential themes which were continuously revised and discussed with co-authors. The original data were re-visited to validate the final themes using an iterative process. ^25^^,^^26^ The analytic process was supported by NVivo version 12 software ^27^ (this can be replicated using Taguette, a free, open source alternative).

## Results

The analysis of the data resulted in the identification of one main theme, ‘wishing to return to the habitual everyday life’, which is expanded on below, including four subthemes. Barriers and facilitators to performing the home-based rehabilitation exercise program were identified throughout the theme and subthemes and are summarized in Table 2.

**Table 2.** Facilitators and barriers to home based rehabilitation exercises.

Facilitators	Barriers
• Goal of returning to everyday life • Flexibility regarding time and place • Obligation due to participation in other research study (PHETHAS-1) and to the associated researcher	• Pain, both absence and presence of pain • Boring exercises • Lack of support from physiotherapist • Being able to perform usual everyday activities

### Wishing to return to the habitual everyday life

All participants wished to be physically active and their overall goal was to return to their habitual everyday life. The following quote came from a male participant who was still working and enjoyed being active through sports.

*P04: The goal was to be able to do sports again. Primarily to be pain free. And then leading a more or less ordinary life again with some sports. […] Aesthetically it is nice to get outside and experience the world with your eyes and ears, as you do when you go outside. First and foremost, the aim of the operation was to get my quality of life back again and then pain free.*

Participants’ goals were to return to their usual everyday life, consisting of activities that contribute to their quality of life. What they perceived as valuable activities were unique to each individual, and they used their own habitual everyday activities as a reference point. Therefore, the primary facilitator for performing the home-based rehabilitation exercise program was the participants' belief that conducting the exercise program would bring them closer to their goal of returning to their everyday life.

When asked whether there were times when it was difficult to get the home-based rehabilitation exercise done, a male participant who worked part time in a shop answered:

*P07: No. If something came up, I did it in the evening. […] I think it is nice you can decide for yourself when you do it, compared to going somewhere to see a physiotherapist.*

Analysis showed that for some participants, flexibility on when and where to include the rehabilitation exercise in their everyday life helped facilitate their performance of rehabilitation exercise. The flexibility made room for prioritizing activities that they considered as contributing to their quality of life, because they could perform the exercise program at a suiting time and place, which also meant that rehabilitation was free of geography, creating a possibility for visiting friends and family without compromising their rehabilitation program. In this sense, the home-based rehabilitation exercises had an advantage compared to supervised rehabilitation.

*Lack of contact to a physiotherapist*

Some participants missed being in contact with a physiotherapist during the rehabilitation period. One participant, who was retired from an office job, described how she took initiative to phone the hospital staff to address certain issues she was worried about. She would have preferred to participate in group training with a physiotherapist compared to performing home-based rehabilitation.

*P12: Because first of all you could have your exercises corrected. Second you could have been told when to use a tighter elastic band. And talk to the others. And this thing in my head being so afraid of crossing my legs, I think that could have been killed [laughing]. And then I might have been able to talk about pain in the groin, because I did have a lot of pain in the groin. Just being told to go see my own [private] physiotherapist with that problem. That would have been nice.*

Lack of contact with a physiotherapist during the period of performing the home-based rehabilitation exercise could be identified as a potential barrier for some participants. The quote above reflects the patient’s concerns about missing the possibility to address issues of uncertainty with both a physiotherapist and other THA patients.

*Presence and absence of pain*

Most participants had experienced post-surgical pain, but in a degree that did not affect their performance of the home-based rehabilitation exercise. However, analysis revealed that both having more intense pain as well as having no pain affected performance of home-based rehabilitation exercise. A male participant working in academia and who was usually active, experienced rather intense pain and described his struggle:

*P06: I think the challenge all along has been how much it must hurt. We are instructed to repeat to exhaustion, […]but where is that point when you are in pain?*

Other participants felt hardly any pain at all. An active female participant, who was retired from the healthcare sector, explained how having no pain affected her:

*P14: When you get out of bed three hours after the surgery and walk and bike and climb stairs and go all the way down the hall and back again and you notice nothing. Then you say to yourself: nothing is wrong with you. […] Then you really have to pull yourself together to do the exercises, because you already feel that you can do everything.*

Our analysis paradoxically showed that both pain and the absence of pain can be seen as barriers in regard to performing the home-based rehabilitation exercise. Experiencing pain lead to insecurity about whether exercises were performed correctly or whether to perform them at all, since pain might be associated with not performing exercises correctly or with exercises being harmful.

Absence of pain made it possible for participants to achieve their goal and return to their habitual and preferred everyday life. Home based rehabilitation exercises were not a part of any participants’ usual everyday life, therefore absence of pain made it tempting to stop conducting the rehabilitation exercise program.

*General physical activity versus rehabilitation exercise*

Participants consistently distinguished between the instructed home-based rehabilitation exercise which was not part of their usual everyday life, and the general physical activities they considered part of their everyday life. Analysis showed no barriers towards general physical activities. Subsequently, analysis identified the subtheme ‘general physical activity versus rehabilitation exercise’.

This retired female participant was usually very active with hiking and fitness. She explained:

*P001: Well, I would rather do normal activities, long walks or something like that. That’s what I prefer. And I do the exercises to achieve that. I mean, it is quite boring doing those exercises, it depends on what’s on the radio [laughs]. […] I do them to be able to do the other things.*

For most participants, the rehabilitation exercise was used as a means of regaining their habitual everyday life. Their usual general physical activities contributed to their understanding of quality of life and were considered joyful, hence they were more motivated to engage in physical activity compared the rehabilitation exercises. But the goal of returning to usual activities became a facilitator for conducting the rehabilitation exercise, even though they were perceived as boring and time consuming.

A male participant, who had already restarted work and exercise, explained why he no longer performed the rehabilitation exercise as instructed.

*P008: It is going so well [laughing]. I do them, [the exercises] but not … maybe not every day, and there are days where I have forgotten.*

Being able to perform usual everyday activities became a barrier for doing the exercise program for some participants, because they already had achieved their goals of returning to their habitual every life. To this group, as their level of functioning improved and they were able to perform their usual activities, they perceived the rehabilitation exercises as having lost their purpose.

*Impact of participation in PHETHAS-1*

Analysis revealed a difference between the group of standard care participants and participants also enrolled in PHETHAS-1. Standard care participants more often modified the home-based rehabilitation exercises as illustrated in the citation above.

In contrast, a very active male participant enrolled in PHETHAS-1 explained his motivation for performing the exercises:

*Interviewer: […] As you have resumed all these usual activities, are you still motivated for doing the exercises with the elastic band?*

*P08: I think so, yes. Absolutely, because it is part of this trial [PHETHAS-1] that I wish to be very loyal to. So I have followed it completely. Otherwise you can’t use it for anything if you don’t know whether one just filled it [the training diary] out as one pleases.*

Analysis showed that for the group of participants enrolled in PHETHAS-1, their enrolment served as a facilitator for performing the rehabilitation exercises exactly as instructed, even after they had resumed their habitual everyday life. Participants referred to an obligation towards the researcher in PHETHAS-1 and the study they had signed up for, and also rationalized, that performing the exercises would be beneficial e.g. in terms of muscle strengthening.

## Discussion

Based on analysis of 22 interviews with participants who had undergone THA surgery we found that participants had a goal of returning to their habitual conduct of everyday life after their THA surgery, and this goal served as a main facilitator for performing the home-based rehabilitation exercise program. Participants' goal of returning to their usual everyday life have also been reported in other studies, ^12^^,^^28^ and a review by Smith et al conclude that patients have little interest in achieving greater levels of physical activity than they had before the hip restricted their functioning. ^27^ Returning to the usual conduct of everyday life is also a well-known motivating force within the theory of critical psychology. ^29^ In that light, it is not surprising that the participants in our study began modifying the exercise program, when they were able to return to their habitual activities.

Data also showed that participants were motivated for general physical activity and would prefer such activities rather than performing the home-based rehabilitation exercise. As general physical activities are part of everyday life, this motivation correlates well with the goal of returning to the habitual everyday life. Meanwhile this strong motivation for reaching their goals, supported participants in overcoming the barrier of perceiving the home-based rehabilitation exercises as boring.

This finding is supported by a study by Specht et al who find that patients are motivated to perform rehabilitation exercises to achieve individual goals. ^30^ Specht et al discuss how patients with intrinsic motivation might be better suited for home-based rehabilitation exercise program, compared to patients with more extrinsic motivation, who might need motivation like obligations towards a professional to perform the exercises. ^30^ In our study, the participants' individual goal was to return to their everyday life. Using the concepts of intrinsic and extrinsic motivation, the goal of returning to their habitual conduct of everyday life would serve as an intrinsic motivation, but this would no longer apply when the goal is reached. So there appears to be an additional temporal factor involved, since the intrinsic motivation of having a goal of returning to the usual everyday life fades when the goal is reached or partly reached. When aiming for adherence towards a home based rehabilitation program for a specific period of time, an initial period of performing the program on their own might advantageously be followed by a period with contact to a physiotherapist. On the other hand, we might want to consider the relevance of aiming for adherence to a rehabilitation exercise program for a specific period of time. If the patient is rehabilitated and have reached their goal of returning to their habitual everyday life earlier than estimated, is performance of rehabilitation exercise still necessary?

Some participants described a wish to have contact with a physiotherapist who would be able to provide guidance and support during the rehabilitation period. Positive impact of being in contact with a therapist during rehabilitation is supported in a review by Davenport et al. ^31^ In terms of motivation this would serve as an extrinsic motivational factor for performing the home based rehabilitation exercises. Some of the participants in this study wished for contact to a physiotherapist, because they experienced barriers such as doubt of whether the exercises were performed correctly or whether to perform them at all e.g. due to pain. In such a situation the person might be fully motivated for performing the exercises on their own, but choose not to do so due to doubt regarding how to handle pain. According to Davenport et al, this would be accommodated when in contact with a therapist. ^31^

Specht et al showed that patients can have a feeling of uncertainty when being left alone to perform the rehabilitation exercise after discharge from the hospital when dealing with pain. ^30^ Our study adds the knowledge that not only pain, but also *absence* of pain can be identified as a barrier for performing home-based rehabilitation exercise. Standard care participants in this study gradually modified the exercises as they were able to return to their habitual everyday life, and performed the usual general physical activities they felt contributed to their quality of life instead. Modifying the exercises might also be a way of handling the fact that most participants described the exercises as boring. Modifying therapeutic instructions is well known in other areas, ^29^ but to our knowledge, this is the first time it is described in THA patients.

The difference found between the two groups regarding adherence towards the home based rehabilitation exercises is also important when reading and assessing results from clinical training studies. Results from our study indicate that participants also enrolled in PHETHAS-1 had a higher degree of adherence in terms of performing exercises exactly as instructed for the full period of time recommended, while participants recruited from usual care modified exercises to a certain degree. Ultimately this would mean that caution should be taken regarding applying results from studies to patients in usual care, since they might not adhere to exercise programs in the same degree as participants in clinical training studies.

### Perspectives

Several perspectives grow out of our study both in terms of implications for practice and for further research.

Importantly, most participants appreciate the home-based rehabilitation exercise program underscoring the flexibility of deciding for themselves when and where to perform the exercises. We have described in detail what this particular rehabilitation exercise program consisted of in Mikkelsen et al 2019. ^13^ Based on this, our study supports the use of home-based rehabilitation exercise, but attention should be paid towards patients who might need additional support and easy access to a physiotherapist should be provided.

Concerning participants wish to return to their usual everyday lives and this serving as a motivational factor for performing the exercises, it is important that healthcare professionals are in dialogue with the individual patient to identify which activities are preferred in their habitual everyday life, and on that basis decide on relevant goals for the rehabilitation period.

Further, physiotherapists working in this area might want to consider how to include general physical exercise into rehabilitation exercise programs, since participants generally favored this type of exercise over formal exercises. This might also address the finding of home-based rehabilitation exercises perceived as boring and thereby increase motivation and support adherence to the exercise program. Additionally, it would be useful to investigate whether general physical activities could be as effective as home-based rehabilitation exercise, and if so, whether future THA patients could rehabilitate by only doing their preferred physical activities.

There may be additional contributing factors in relation to patients’ perceptions of home-based rehabilitation exercise after THA. These could include age, gender, previous training experience, and culture. Further studies are needed to explore this.

### Strengths and limitations

In this study we recruited participants from both the PHETHAS-1 study and from standard care. This combination of participants revealed an important difference in motivation towards adherence to, and performance of, the home-based rehabilitation program. The PHETHAS-1 participants had a higher degree of motivation towards adherence over time and performing exercises as instructed, but our findings showed no other differences between the two groups of participants. In addition to this specific point, one might argue that findings from the PHETHAS-1 participants have a lower degree of transferability to practice compared to the usual care participants. The two groups of participants also differed regarding the location of the interviews. Participants recruited from usual care were interviewed in their homes, while participants recruited from PHETHAS-1 study were interviewed in a hospital setting. This might have affected data e.g. if participants were more comfortable in their homes as well as being in the setting where the home based rehabilitation exercises were performed and therefore would give more open hearted and detailed interviews. Data retrieved from interviews conducted in both types of settings are similar which indicate that this potential limitation is not applicable to this study.

We recruited participants from only one hospital, which might affect transferability. Since the rehabilitation after THA differs between hospitals we have described in detail what this particular rehabilitation consisted of.

The participants in our study are considered relatively physically active, which could have influenced the results and it would have been favorable to have included more sedentary patients as well. Additionally, our participants consisted of fewer females which differs from the group of patients undergoing THA in general, where more females undergo THA compared to males. This might have affected our results.

In this study the participants did not expressed any barriers towards general physical activity. This might be a result of the patients considering barriers as only relevant towards activities they are expected to be able to perform, like the rehabilitation exercises, compared to activities they wish to be able to perform at some point in time. Also, in retrospective, the interview guide primarily focused on barriers towards performing the rehabilitation exercises and it might have enhanced nuances in data regarding potential barriers, if the interview guide had included specific questions regarding potential barriers toward general physical activity.

Scientific rigor is enhanced in this study by using theory throughout the scientific process ^32^^,^^33^ and triangulation in the form of more investigators collaborating on the analysis, is also considered a strength. ^33^

## Conclusion

The study findings showed that returning to habitual everyday life was a common goal for patients rehabilitating after THA. Thereby, habitual everyday life became a crucial mental driving force during the rehabilitation period and served as a facilitator for performing the home-based rehabilitation exercise, which was perceived as a means to achieve their goal. Attention should be paid towards each patient’s facilitators and barriers for home-based rehabilitation exercise, and it seems especially important to identify each individual's preferred activities in their habitual everyday life when planning and setting relevant goals for the rehabilitation period.

## Data availability

### Underlying data

Access to this data is restricted due to ethical reasons. The data cannot be made publicly available as it is not possible to sufficiently de-identify the interview transcripts, which contain information that could compromise research participant privacy and consent. Transcripts can be made available upon reasonable request e.g., for the purpose of reviewing this article. Please contact the corresponding author, Anne Grøndahl Poulsen ( anngrora@rm.dk). Please note that transcripts are in Danish.

### Extended data

Figshare: PHETHAS-2. https://doi.org/10.6084/m9.figshare.14101877.v2. ^22^

This project contains the following extended data:

• Interviewguide.docx (semi-structured interview guide).

### Reporting guidelines

Figshare: COREQ checklist for ‘Patient perspectives on home-based rehabilitation exercise and general physical activity after total hip arthroplasty: A qualitative study (PHETHAS-2)’. https://doi.org/10.6084/m9.figshare.14101877.v2. ^22^

Data are available under the terms of the Creative Commons Attribution 4.0 International license (CC-BY 4.0).
