# Supplementary material for: Patient perspectives on home-based rehabilitation exercise and general physical activity after total hip arthroplasty: A qualitative study (PHETHAS-2)
Source: F1000Res. 2023 Jun 26;10:382. Originally published 2021 May 13. [Version 5] doi: 10.12688/f1000research.51684.5 (PMC10285331; doi:10.12688/f1000research.51684.5)
Supplement: Peer review history

# Peer review: Reviewer response for version 5

**DOI:** 10.5256/f1000research.151910.r181572

**Published:** 2023-07-14

**Authors:** Thomas W Wainwright, Louise Mew

**Affiliations:** 1 Orthopaedic Research Institute, Bournemouth University, Poole, England, UK; 2 University Hospitals Dorset NHS Foundation Trust, Poole, England, UK

Thank you for making the suggested changes.

Is the work clearly and accurately presented and does it cite the current literature?

Yes

If applicable, is the statistical analysis and its interpretation appropriate?

Not applicable

Are all the source data underlying the results available to ensure full reproducibility?

Yes

Is the study design appropriate and is the work technically sound?

Yes

Are the conclusions drawn adequately supported by the results?

Yes

Are sufficient details of methods and analysis provided to allow replication by others?

Yes

Reviewer Expertise:

Orthopaedic surgery, physiotherapy, nursing, rehabilitation, enhanced recovery after surgery

We confirm that we have read this submission and believe that we have an appropriate level of expertise to confirm that it is of an acceptable scientific standard.

# Peer review: Reviewer response for version 4

**DOI:** 10.5256/f1000research.136997.r175917

**Published:** 2023-06-16

**Authors:** Thomas W Wainwright, Louise Mew

**Affiliations:** 1 Orthopaedic Research Institute, Bournemouth University, Poole, England, UK; 2 University Hospitals Dorset NHS Foundation Trust, Poole, England, UK

This is a well-conducted and good study write-up, which is reported transparently and balanced in its conclusions. The qualitative approach is well chosen and allows the patient's perspective to be described. The authors have responded well to previous reviewers' recommendations, and these changes have improved the article. 

 We have a couple of minor points for the authors to consider.

1. For clarity, please could the authors list the four sub-themes in the second line of the results section after they state there are four sub-themes. 
2. The findings regarding pain (presence and absence) and its relation to performing exercises are particularly interesting and highlighted in the abstract's conclusion. This is a novel angle and a perspective that has come from the patient's perspective, however, we feel that this finding could be explored and considered in more detail in the discussion section. 

Is the work clearly and accurately presented and does it cite the current literature?

Yes

If applicable, is the statistical analysis and its interpretation appropriate?

Not applicable

Are all the source data underlying the results available to ensure full reproducibility?

Yes

Is the study design appropriate and is the work technically sound?

Yes

Are the conclusions drawn adequately supported by the results?

Yes

Are sufficient details of methods and analysis provided to allow replication by others?

Yes

Reviewer Expertise:

Orthopaedic surgery, physiotherapy, nursing, rehabilitation, enhanced recovery after surgery

We confirm that we have read this submission and believe that we have an appropriate level of expertise to confirm that it is of an acceptable scientific standard.

# Peer review: Reviewer response for version 4

**DOI:** 10.5256/f1000research.136997.r146561

**Published:** 2022-08-09

**Authors:** Brocha Z. Stern

**Affiliations:** 1 Icahn School of Medicine at Mount Sinai, New York, NY, USA

Thank you for the revisions to this very interesting manuscript on a timely and meaningful topic. I have no further comments.

Is the work clearly and accurately presented and does it cite the current literature?

Partly

If applicable, is the statistical analysis and its interpretation appropriate?

Not applicable

Are all the source data underlying the results available to ensure full reproducibility?

Partly

Is the study design appropriate and is the work technically sound?

Yes

Are the conclusions drawn adequately supported by the results?

Yes

Are sufficient details of methods and analysis provided to allow replication by others?

Yes

Reviewer Expertise:

Rehabilitation, Qualitative Research, Musculoskeletal Conditions

I confirm that I have read this submission and believe that I have an appropriate level of expertise to confirm that it is of an acceptable scientific standard.

# Peer review: Reviewer response for version 3

**DOI:** 10.5256/f1000research.135426.r142817

**Published:** 2022-07-05

**Authors:** Brocha Z. Stern

**Affiliations:** 1 Icahn School of Medicine at Mount Sinai, New York, NY, USA

Thank you for the opportunity to review this revision. The authors have thoughtfully reflected on and addressed previous comments. This already interesting manuscript is much richer and clearer now.

 Of note, the abstract (specifically the results) was not updated in this revision, and the number of themes/subthemes in the abstract is not consistent with the text.

 The findings are much stronger. But - visually - it is unclear how the themes and subthemes are broken up. The formatting makes it appear that there are two primary themes while the authors state there is one theme and four subthemes.

 Linkage of the barriers/facilitators in Table 2 to the new subthemes could also be clearer. Reframing the first paragraph of the Results section may help, e.g.: “The analysis of the data resulted in the identification of one main theme, ‘wishing to return to the habitual everyday life’, which is expanded on below, including four subthemes. Barriers and facilitators to performing the home-based rehabilitation exercise program were identified throughout the theme and subthemes and are summarized in Table 2.”

Is the work clearly and accurately presented and does it cite the current literature?

Partly

If applicable, is the statistical analysis and its interpretation appropriate?

Not applicable

Are all the source data underlying the results available to ensure full reproducibility?

Partly

Is the study design appropriate and is the work technically sound?

Yes

Are the conclusions drawn adequately supported by the results?

Yes

Are sufficient details of methods and analysis provided to allow replication by others?

Yes

Reviewer Expertise:

Rehabilitation, Qualitative Research, Musculoskeletal Conditions

I confirm that I have read this submission and believe that I have an appropriate level of expertise to confirm that it is of an acceptable scientific standard, however I have significant reservations, as outlined above.

# Response

**Published:** 2022-08-01

**Authors:** Anne Poulsen

**Affiliations:** Central Region, Denmark

Dear reviewer

 Thank you for your time and effort spend on reviewing the latest version of the manuscript *Patient perspectives on home-based rehabilitation exercise and general physical activity after total hip arthroplasty: A qualitative study (PHETHAS-2). *Your comments help improve the manusript and clarify areas of importance.

 Please find listed below reviewer comments and our responses including subsequent actions taken to improve the manuscript. We hope you will find that the revisions address the issues commented in the review.

 Sincerely,

 Anne Grøndahl Poulsen, on behalf of the authors

**Comment 1**: *"The abstract (specifically the results) was not updated in this revision, and the number of themes/subthemes in the abstract is not consistent with the text."*

**Response:** Thank for pointing our attention towards this inconsistency between abstract and Results section. We have edited the Abstract to fit the editions made in the Results section in version 3 of the manuscript.

**Comment 2**: *"The findings are much stronger. But - visually - it is unclear how the themes and subthemes are broken up. The formatting makes it appear that there are two primary themes while the authors state there is one theme and four subthemes."*

**Response**: Thank you for noticing that the headings do not support the presentation of our results. The formatting of headings have been edited.

**Comment 3**: *"Linkage of the barriers/facilitators in Table 2 to the new subthemes could also be clearer. Reframing the first paragraph of the Results section may help, e.g.: “The analysis of the data resulted in the identification of one main theme, ‘wishing to return to the habitual everyday life’, which is expanded on below, including four subthemes. Barriers and facilitators to performing the home-based rehabilitation exercise program were identified throughout the theme and subthemes and are summarized in Table 2.”"*

**Response**: Thank you for this suggestion. To minimize any unclarities we have reworded the first paragraph of the Results section as suggested.

# Peer review: Reviewer response for version 2

**DOI:** 10.5256/f1000research.120752.r136480

**Published:** 2022-05-12

**Authors:** Brocha Z. Stern

**Affiliations:** 1 Icahn School of Medicine at Mount Sinai, New York, NY, USA

Thank you for the opportunity to review this paper. This is a qualitative study exploring patients’ perspectives of unsupervised home-based rehabilitation exercise and general physical activity after a total hip arthroplasty. The sample includes patients enrolled in a pragmatic cohort study of unsupervised home-based exercise and patients not enrolled in the cohort study. All qualitative participants received the same exercise instructions and were interviewed at 10 weeks post-surgery. The data were synthesized into one main theme and a subtheme.

 Overall, this is a very interesting paper on a timely topic, and the methods are detailed and transparent. The findings have implications for clinical practice to support patients’ recovery after total hip arthroplasty. The study is strengthened by use of a theoretical concept and inclusion of participants external to the cohort study. Figure 1 is an excellent diagram that helps the reader understand the flow of the study procedures. The discussion has been significantly enhanced in this version of the manuscript. Additionally, the authors engage in thoughtful reflection on whether adherence to rehabilitation exercise is necessary if people have returned to everyday life. Degree of data availability is appropriate given the study design.

 I have a few questions and suggestions to further improve the clarity of reporting and richness of the findings.

**Introduction**

- Additional specificity in the study aim is suggested to clarify that “home-based rehabilitation exercise” refers to an unsupervised program. Otherwise, in certain contexts, this label could be misinterpreted as supervised rehabilitation performed in a home setting versus an outpatient clinic.
- The last sentence in the 3 ^rd^ paragraph may benefit from explicit mention of facilitators and barriers to improve the link between patient perspectives of rehabilitation exercise and the authors’ discussion of adherence.
- The motivation to study patient perspectives of general physical activity, in addition to rehabilitation exercise, should be more clearly specified in the introduction, (particularly given the embedding of the interview study within a cohort study of rehabilitation exercise).

**Methods**

- The definition of “home-based rehabilitation exercise” should perhaps also define “home-based.” Alternatively, the sentence could read, “We defined rehabilitation exercise as a plan of physical activities…”.
- The authors have not fully addressed the previous reviewer’s comments about sampling. The authors confirm in their response that the size was not determined a priori, but how did they determine when to stop interviewing? Was any form of saturation used? Additionally, the modified sentence about purposeful sampling based on the ongoing analysis is unclear. Were specific participants recruited to fill gaps in knowledge (e.g., someone who was very adherent was purposefully selected because of emerging findings related to nonadherence)? Or is this statement actually meant to reflect decisions about how many participants to recruit versus who to recruit (i.e., sample size versus purposeful sampling)?
- Did the standard care participants also have an exercise diary? It is included in the interview guide but only specified in Figure 1 for the PHETHAS-1 participants. If this was only for the PHETHAS-1 participants, could this have influenced the findings in any way, including the increased adherence noted in the cohort subsample?
- Why was the time frame of 10 weeks post-op chosen for the interview? Was this related to how long patients were supposed to perform the home-based program?
- Do AG, JG, or CR have any experience with rehabilitation? If so, that should be noted in addition to their research experience. If not, whether this is a limitation should be considered.
- The data analysis section would be strengthened by additional detail about how the investigators moved from codes to themes.

**Results**

- I have the same comment as the first reviewer regarding the findings. I appreciate the authors’ response that this synthesis best reflected the participants’ experiences in relation to the theoretical concept of “conduct of everyday life.” However, one theme with one subtheme may suggest a relatively shallow analysis given the amount of data collected and the inclusion of inductive coding. Specifically, additional subthemes may have enhanced the clarity and richness of the findings. Currently, a lot of information is distilled into the single main theme, which also limits the cohesion of the findings. For example, some of the specific barriers and facilitators to exercise performance do not directly align with the motivation of returning to everyday life and may have been better synthesized in a subtheme.
- Regarding the label for the main theme, the meaning of “well-known” is unclear and is not evident in the description of the theme.
- In Table 2, the language of “reaching goal of returning to everyday life” may imply that having achieved the goal is the facilitator. I believe the authors actually mean that the “goal of returning to everyday life” is the facilitator.
- In Table 2, what is meant by the obligation to the associated physiotherapist? Wouldn’t this be relevant also for the non-cohort participants as a physio instructed them in their exercises? Is this more specifically obligation to the researcher?
- I find it surprising that participants expressed no barriers toward general physical activities. Wouldn’t the presence of pain, for example, impede? Were there perhaps fewer barriers expressed versus no barriers? Or could this be an artifact related to the interview guide, which focused predominantly on rehabilitation exercise?
- The information about different perspectives in PHETHAS-1 versus standard care participants does not clearly align with the subtheme and may fit better in the main theme. Additionally, regarding the comment that PHETHAS-1 participants shared that the exercises would benefit them personally, the implications are unclear. Are the authors saying that PHETHAS-1 participants perceived more benefit from the exercises because the exercises were situated in a research context?

**Discussion**

- In the perspectives section, a citation to the protocol paper should be included by “we have described in detail what this particular rehabilitation consisted of” as most of the detail is not in the current paper.

**Conclusion**

- The conclusion repeats the detailed findings but would be strengthened by including a general take-home message for the reader.

Is the work clearly and accurately presented and does it cite the current literature?

Partly

If applicable, is the statistical analysis and its interpretation appropriate?

Not applicable

Are all the source data underlying the results available to ensure full reproducibility?

Partly

Is the study design appropriate and is the work technically sound?

Yes

Are the conclusions drawn adequately supported by the results?

Yes

Are sufficient details of methods and analysis provided to allow replication by others?

Yes

Reviewer Expertise:

Rehabilitation, Qualitative Research, Musculoskeletal Conditions

I confirm that I have read this submission and believe that I have an appropriate level of expertise to confirm that it is of an acceptable scientific standard, however I have significant reservations, as outlined above.

# Response

**Published:** 2022-06-22

**Authors:** Anne Poulsen

**Affiliations:** Central Region, Denmark

                                                                                       Silkeborg, Denmark, 22th June 2022

 Dear reviewer,

 Thank you for the thorough review and highly valuable comments, improving the quality of the manuscript *Patient perspectives on home-based rehabilitation exercise and general physical activity after total hip arthroplasty: A qualitative study (PHETHAS-2).*

 Please find listed below the reviewer comments, our responses and subsequent actions taken to improve the manuscript. We find that working with your comments greatly improved the manuscript and we hope you will find that the revisions address the concerns and issues raised in the review.

 Sincerely,

 Anne Grøndahl Poulsen, on behalf of the authors

**Introduction**

Reviewer comment 1

*"Additional specificity in the study aim is suggested to clarify that “home-based rehabilitation exercise” refers to an unsupervised program. Otherwise, in certain contexts, this label could be misinterpreted as supervised rehabilitation performed in a home setting versus an outpatient clinic."*

Author answer

 Thank you for giving notice to this detail which might give rise to confusion. Since patients receive supervision in relation to instructions in the exercise program, we have clarified that the *performance* of the program is unsupervised.

 We have added the passage marked in bold letters:

 The PHETHAS studies were founded to support and optimize clinical pathways with patients rehabilitating at home after THA. PHETHAS-1 ( ClinicalTrials.gov NCT03109821, April 12, 2017) quantitatively investigates the physical outcomes of performing a home-based rehabilitation exercise program , **which after initial instruction was performed without further supervision** **^12^** **, while this study, PHETHAS-2, qualitatively investigates how patients perceive this home-based rehabilitation exercise and general physical activity after THA, focusing on facilitators and barriers.**

Reviewer comment 2

*"The last sentence in the 3 ^rd^ paragraph may benefit from explicit mention of facilitators and barriers to improve the link between patient perspectives of rehabilitation exercise and the authors’ discussion of adherence."*

Author answer:

 This is a relevant suggestion, which we comply with.

 We have added the passage marked in bold letters:

 Yet we know little about patients' perspectives **including facilitators and barriers regarding** home-based rehabilitation exercise and general physical activity after THA.

Reviewer comment 3

*"The motivation to study patient perspectives of general physical activity, in addition to rehabilitation exercise, should be more clearly specified in the introduction, (particularly given the embedding of the interview study within a cohort study of rehabilitation exercise)."*

Author answer:

 Also a relevant suggestion, which we comply with.

 We have added the passage marked in bold letters:

**Studies also find that patients have low expectations to obtain greater levels of activity than pre-operatively ^12^**, yet we know little about patients' perspectives, including facilitators and barriers, regarding home-based rehabilitation exercise and general physical activity after THA.

**Methods**

Reviewer comment 4

*"The definition of “home-based rehabilitation exercise” should perhaps also define “home-based.” Alternatively, the sentence could read, “We defined rehabilitation exercise as a plan of physical activities…”."*

Author answer:

 Thank you for giving notice to this detail which might give rise to confusion. We have added a clarification to the definition. See also response to reviewer comment #1.

 We have added the passage marked in bold letters:

 We defined home-based rehabilitation exercise as a plan of physical activities **in which patients received an initial instructed at the hospital and was performed at the participants' home without further supervision. It is **designed and prescribed to meet specific therapeutic goals.

Reviewer comment 5

*"The authors have not fully addressed the previous reviewer’s comments about sampling. The authors confirm in their response that the size was not determined a priori, but how did they determine when to stop interviewing? Was any form of saturation used?*

*Additionally, the modified sentence about purposeful sampling based on the ongoing analysis is unclear. Were specific participants recruited to fill gaps in knowledge (e.g., someone who was very adherent was purposefully selected because of emerging findings related to nonadherence)? Or is this statement actually meant to reflect decisions about how many participants to recruit versus who to recruit (i.e., sample size versus purposeful sampling)?"*

Author answer:

 Thank for this comment. We have added further details regarding the sampling process, describing the information we had to base our sampling of possible participants on, and how obtained quality of data guided termination of sampling.

 We have edited the passage marked in bold letters:

 The participants were purposely sampled **with the aim of reflecting the gender and ages of typical THA patients as well as representation of both patients from standard care and patients participating in Phethas-1 (see below). Furthermore, sampling was based on the ongoing analysis including themes that were under continual development.** **When thick descriptions of the themes were obtained and no new themes were identified,** **recruitment was terminated**. All invited patients accepted participation.

Reviewer comment 6

*"Did the standard care participants also have an exercise diary? It is included in the interview guide but only specified in Figure 1 for the PHETHAS-1 participants. If this was only for the PHETHAS-1 participants, could this have influenced the findings in any way, including the increased adherence noted in the cohort subsample?"*

Author answer:

 It is correct, that the exercise diary was part of PHETHAS-1 and therefore standard care participants did not have an exercise dairy. It is a good point, and we have added a sentence relating to this comment in the Discussion section.

 We have added the following sentence to the Discussion section:

 Furthermore, it could be speculated that outcome assessments in PHETHAS-1 (exercise dairy, elastic band sensor and physical testing) may increase adherence for participants compared to standard care patients, who did not have a sensor on their elastic band, an exercise diary, nor were they physically tested.

Reviewer comment 7

*"Why was the time frame of 10 weeks post-op chosen for the interview? Was this related to how long patients were supposed to perform the home-based program?"*

Author answer:

 PHETAS-1 was designed as a pragmatic study and many of the deign choices reflect this approach. We wanted the patients to have had sufficient experience in performing home-based rehabilitation exercise after their surgery. We thought 10 weeks would be sufficient. Ten weeks was also the time point where the participants came in for their assessment of the quantitative outcomes. So, to reduce the participatory burden and increase study efficiency, we conducted the interviews at 10 weeks.

 We have added the following sentence to the Data collection section:

 At this point in time patients had experience performing the home-based exercise program and 10 weeks postoperatively was also the point of time for the physical testing of PHETHAS-1 participants and thereby the participatory burden for these participants was minimized.

Reviewer comment 8

 " *Do AG, JG, or CR have any experience with rehabilitation? If so, that should be noted in addition to their research experience. If not, whether this is a limitation should be considered*."

Author answer:

 Thank for this relevant suggestion which we have addressed.

 We have added the following sentence to the Methods section:

 AG is a physiotherapist with experience in rehabilitation following THA and both JG and CR are registered nurses without specific experience with rehabilitation following THA.

Reviewer comment 9

*"The data analysis section would be strengthened by additional detail about how the investigators moved from codes to themes."*

Author answer:

 Thank you for commenting on how detailed the analysis is described.

 We have added the passage marked in bold letters:

 The inductive part of analysis added an openness for other themes of importance in the dataset. **The coding process generated potential themes, which were continuously revised and discussed with co-authors using an iterative process**. The original data were re-visited to validate the final themes.

**Results**

Reviewer comment 10

*"I have the same comment as the first reviewer regarding the findings. I appreciate the authors’ response that this synthesis best reflected the participants’ experiences in relation to the theoretical concept of “conduct of everyday life.” However, one theme with one subtheme may suggest a relatively shallow analysis given the amount of data collected and the inclusion of inductive coding. Specifically, additional subthemes may have enhanced the clarity and richness of the findings. Currently, a lot of information is distilled into the single main theme, which also limits the cohesion of the findings. For example, some of the specific barriers and facilitators to exercise performance do not directly align with the motivation of returning to everyday life and may have been better synthesized in a subtheme."*

Author answer:

 Thank you. We fully agree that especially the main theme has many nuances and details. We have chosen to meet the suggestion by dividing the section into additional subthemes, to support clarity and underscore the many nuances the themes included.

 We have restructured the results section, which is now presented with one main theme and four subthemes to better illustrate the nuances and enhance clarity in our findings as commented on.

Reviewer comment 11

*"Regarding the label for the main theme, the meaning of “well-known” is unclear and is not evident in the description of the theme."*

Author answer:

 Thank for this comment, which we have addressed.

 The term "well-known" is replaced with habitual throughout the paper.

Reviewer comment 12

*"In Table 2, the language of “reaching goal of returning to everyday life” may imply that having achieved the goal is the facilitator. I believe the authors actually mean that the “goal of returning to everyday life” is the facilitator."*

Author answer:

 Thank for this relevant suggestion which we have addressed.

 We have edited the sentence into:

 "goal of returning to everyday life”

Reviewer comment 13

*"In Table 2, what is meant by the obligation to the associated physiotherapist? Wouldn’t this be relevant also for the non-cohort participants as a physio instructed them in their exercises? Is this more specifically obligation to the researcher?"*

Author answer:

 Thank for this comment, which we have addressed.

 Associated physiotherapist is replaced with **associated researcher**.

Reviewer comment 14

*"I find it surprising that participants expressed no barriers toward general physical activities. Wouldn’t the presence of pain, for example, impede? Were there perhaps fewer barriers expressed versus no barriers? Or could this be an artifact related to the interview guide, which focused predominantly on rehabilitation exercise?"*

Author answer:

 Thank you for this comment. It is a good point which we have addressed by adding a passage to the Discussion section.

 We have added the following passage to the Discussion section:

 In this study the participants did not expressed any barriers towards general physical activity. This might be a result of the patients considering barriers as only relevant towards activities they are expected to be able to perform, like the rehabilitation exercises, compared to activities they wish to be able to perform at some point in time. Also, in retrospect, the interview guide primarily focused on barriers towards performing the rehabilitation exercises and it might have enhanced nuances in data regarding potential barriers, if the interview guide had included specific questions regarding potential barriers toward general physical activity.

Reviewer comment 15

*"a) The information about different perspectives in PHETHAS-1 versus standard care participants does not clearly align with the subtheme and may fit better in the main theme.*

*b) Additionally, regarding the comment that PHETHAS-1 participants shared that the exercises would benefit them personally, the implications are unclear. Are the authors saying that PHETHAS-1 participants perceived more benefit from the exercises because the exercises were situated in a research context?"*

Author answer:

 Thank you for these comments.

 a) We have restructured the results section, which is now presented with one main theme and four subthemes, including the section referred to in this comment.

 See also reviewer comment #10.

 b) We have rephrased the commented sentence into:

 Analysis showed that for the group of participants enrolled in PHETHAS-1, their enrolment served as a facilitator for performing the rehabilitation exercises exactly as instructed, **even after they had resumed their** **habitual everyday life.**  Participants referred to an obligation towards the researcher in PHETHAS-1 and the study they had signed up for, **and also rationalized, that performing the exercises would be beneficial e.g. in terms of muscle strengthening.**

**Discussion**

Reviewer comment 16

*"In the perspectives section, a citation to the protocol paper should be included by “we have described in detail what this particular rehabilitation consisted of” as most of the detail is not in the current paper."*

Author answer

 Thank for this relevant suggestion which we have addressed.

 We have added the following sentence and reference to the protocol paper:

 We have described in detail what this particular rehabilitation exercise program consisted of in Mikkelsen et al 2019 ^1^ ^3^.

**Conclusion**

Reviewer comment 17

*"The conclusion repeats the detailed findings but would be strengthened by including a general take-home message for the reader."*

Author answer:

 Thank you for the suggestion. We have edited the conclusion.

 We have rephrased the conclusion:

 The study findings showed that returning to habitual everyday life was a common goal for patients rehabilitating after THA. Thereby, habitual everyday life became a crucial mental driving force during the rehabilitation period and served as a facilitator for performing the home-based rehabilitation exercise, which was perceived as a means to achieve their goal. Attention should be paid towards each patient's facilitators and barriers for home-based rehabilitation exercise, and it seems especially important to identify each individual's preferred activities in their habitual everyday life when planning and setting relevant goals for the rehabilitation period.                                                                                                             

# Peer review: Reviewer response for version 1

**DOI:** 10.5256/f1000research.54876.r85349

**Published:** 2021-06-17

**Authors:** Kirsten Specht

**Affiliations:** 1 Department of Orthopaedic Surgery, Hospital Sønderjylland, University Hospital of Southern Denmark, Aabenraa, Denmark; 2 Department of Regional Health Research, University of Southern Denmark, Odense, Denmark

This article addresses patients’ perspectives on home-based rehabilitation exercises following total hip arthroplasty (THA). The topic is investigated through a qualitative method using semi-structured interviews. Data were analysed using thematic analysis. The study found the theme: 'wishing to return to the well-known everyday life', and the subtheme 'general physical activity versus rehabilitation exercise'. The topic of the paper is interesting and overall the paper is well-written; however, there are a number of issues throughout the paper.

**Introduction:**

- The Introduction is a well-written section uncovering the knowledge within THA and home-based rehabilitation exercise.

**Aim:**

- There are some concerns about the aim as it could reflect the title in a better way. The semi-structured interview guide sets the stage for broader and more open interviews with the patients’ perspectives on the topic in general, including facilitators and barriers. Rewording is wanted for the aim so that it better reflects the title which has a more open approach. A suggestion could be something like this: 'the study aimed to investigate how patients perceived home-based rehabilitation exercise and general physical activity after THA, including facilitators and barriers'.

**Methods:**

- The theoretical underpinning with the use of the concept 'conduct of everyday life' fits the topic very well, and the definition of home-based rehabilitation exercise is helpful for the overall understanding of the paper.
- The study investigates home-based rehabilitation exercises, for international readers, it is crucial to understand what it contains in Denmark and Silkeborg. Therefore, there is a need for a detailed description of home-based rehabilitation exercises.
- Furthermore, was it the same programme for the two included groups? The authors have reference number 10 to describe the home-based rehabilitation exercise, this is a review, which confuses me. Please elaborate on the details of the programmes and whether they were similar for the two groups.

Regarding the patients and recruitment, please elaborate on some of the details:

- Sample size – how was that determined?
- How many were asked to participate in the study?
- Did any refuse to participate?
- Included participants from the PHETHAS-1 study were 14 – how many were included in the PHETHAS-1 study, as this might be the group of patients where the participants in the PHETHAS-2 study were selected?

**Results:**

- Taking into account that the interviews lasted an average of 43 minutes and the open questions in the semi-structured interview guide, it is surprising that only one theme, including one under-theme, was generated. There must be huge data material from all these minutes of interviews. During the analysis process, could it be possible to find more valuable results to give more perspectives on the aim?
- Quote P12: “Because first of all…" – Could another interpretation be more like a desire for a contact to the healthcare system because of uncertainty?
- To build upon what is already known, it could be interesting if there were any quotes about the last question in the interview guide: We are considering investigating whether this exercise program works better than general physical activities, what do you think of this idea?

**Discussion:**

- The Discussion section is very short. Further discussion about the meaning of motivation, intrinsic and extrinsic factors could be valuable for the paper.
- The concept 'conduct of everyday life' was chosen as a theoretical framework, it would have been interesting to bring this framework into the discussion. Additionally, topics such as pain and absence of pain and individual expectations alignment could be further discussed.
- To improve the Discussion section, more research and other theories on, for example, the above-mentioned topics could expand on the results and make implications for practice more clear.
- Patients from the two groups were interviewed in different places (at the hospital and in their homes), what impact could that have on the results? In addition, patients being a part of another study, what consequences does that have for the transferability of the study?
- Two paragraphs in the Discussion section belong more to the Conclusion or a perspective section: “Overall the participants …” and “There may be additional …”.

**Conclusion:**

- The conclusion is supported by the results. However, adding a perspective section would be helpful to clarify how the findings can improve clinical practice."

Is the work clearly and accurately presented and does it cite the current literature?

Partly

If applicable, is the statistical analysis and its interpretation appropriate?

Not applicable

Are all the source data underlying the results available to ensure full reproducibility?

No source data required

Is the study design appropriate and is the work technically sound?

Yes

Are the conclusions drawn adequately supported by the results?

Yes

Are sufficient details of methods and analysis provided to allow replication by others?

No

Reviewer Expertise:

Orthopaedics, fast-track in hip and knee arthroplasty, qualitative studies

I confirm that I have read this submission and believe that I have an appropriate level of expertise to confirm that it is of an acceptable scientific standard, however I have significant reservations, as outlined above.

# Response

**Published:** 2022-01-27

**Authors:** Anne Poulsen

**Affiliations:** Central Region, Denmark

Dear Reviewer,

 Thank you for the thorough review and highly valuable comments, improving the quality of the manuscript *Patient perspectives on home-based rehabilitation exercise and general physical activity after total hip arthroplasty: A qualitative study (PHETHAS-2).*

 Please find listed below the reviewer comments, our responses, and subsequent actions taken to improve the manuscript. We find that working with your comments greatly improved the manuscript and we hope you will find that the revisions address the concerns and issues raised in the review.

 Sincerely,

 Anne Grøndahl Poulsen, on behalf of the authors

 No 1.

 Reviewer comments:

 There are some concerns about the aim as it could reflect the title in a better way. The semi-structured interview guide sets the stage for broader and more open interviews with the patients’ perspectives on the topic in general, including facilitators and barriers. Rewording is wanted for the aim so that it better reflects the title which has a more open approach. A suggestion could be something like this: 'the study aimed to investigate how patients perceived home-based rehabilitation exercise and general physical activity after THA, including facilitators and barriers'.

 Author response:

 Thank you for turning our attention towards a better coherence between title and aim.

 We have taken your suggestion into consideration and reworded the aim. At the same time (for transparency), we want to stay true to the meaning and wording of the original aim as laid out in the trial protocol (see reference under No.2, Action taken). We feel that the current revision of the aim does that.  

**

 Action taken:

 Rewording of the aim into:

*To investigate how patients perceived home-based rehabilitation exercise and general physical activity after THA, focusing on facilitators and barriers.*

 No 2.

 Reviewer comments:

 The study investigates home-based rehabilitation exercises, for international readers, it is crucial to understand what it contains in Denmark and Silkeborg. Therefore, there is a need for a detailed description of home-based rehabilitation exercises.

 Furthermore, was it the same programme for the two included groups? The authors have reference number 10 to describe the home-based rehabilitation exercise, this is a review, which confuses me. Please elaborate on the details of the programmes and whether they were similar for the two groups.

 Author response:

 Thank you for making this point and for your sharp observation towards the wrongly noted reference. We have addressed this comment in two ways.

 A) Correcting the reference in which a full description of the home-based rehabilitation exercises is included.

   

 B) The article already states that the two groups were instructed in the exact same rehabilitation exercises. As a supplement, we have added a passage that describes the rehabilitation exercises in brief terms.

 Action taken:

 A) Correction of reference into:

 Mikkelsen LR, Madsen MN, Rathleff MS, Thorborg K, Rossen CB, Kallemose T, et al. Pragmatic Home-Based Exercise after Total Hip Arthroplasty-Silkeborg: Protocol for a prospective cohort study (PHETHAS-1) 2019.

 B) The following passage is added:

*This included an instruction* *before hospital discharge, where the patients were instructed to perform daily unloaded hip exercises for the first three weeks. At the clinical follow up after three weeks, the patients were instructed to perform strengthening exercises every second day, supplemented with daily balance exercise and stretching of the hip flexor muscles. The strengthening exercises were hip abduction, hip flexion and hip extension with elastic band resistance plus sit-to-stand exercise.*

 No 3.

 Reviewer comments:

 Regarding the patients and recruitment, please elaborate on some of the details:

 Sample size – how was that determined?

 How many were asked to participate in the study? Did any refuse to participate?

 Author response:

 Thank you for these comments regarding the sampling process. The sampling process followed the principles of purposeful sampling and hence the number of participants was not determined ahead of recruitment. We have elaborated the passage describing the recruitment process in more detail and hope it will answer the questions asked.

 Action taken:

 The following passage in italics is added:

*The participants were purposely sampled hence sampling was based on the ongoing analysis including themes that were under continual development and with the aim of reflecting the gender and ages of typical THA patients. All invited patients accepted participation.*

 No 4.

 Reviewer comments:

 Included participants from the PHETHAS-1 study were 14 – how many were included in the PHETHAS-1 study, as this might be the group of patients where the participants in the PHETHAS-2 study were selected?

 Author response:

 Thank you for this comment. The number of participants in the PHETHAS-1 study is 94. This will of course be stated when the PHETHAS-1 study is published. It is unclear to us how this information could be relevant for the qualitative PHETHAS-2 study. Please do not hesitate to comment on this again and elaborate if you find it needed.

 Action taken:

 No action taken.

 No 5.

 Reviewer comments:

 Taking into account that the interviews lasted an average of 43 minutes and the open questions in the semi-structured interview guide, it is surprising that only one theme, including one under-theme, was generated. There must be huge data material from all these minutes of interviews. During the analysis process, could it be possible to find more valuable results to give more perspectives on the aim?

 Author response:

 Thank you for this perspective on the relationship between the length of interviews and identified themes. We find the content of the two themes reflects the content of the interviews related to the concept of everyday life, which is a comprehensive concept. In relation to the aim, the identified themes do provide several aspects of the focus on barriers and facilitators towards performing home-based rehabilitation exercises, which might not be stated very clearly. Therefore, we have added a table illustrating facilitators and barriers to home-based rehabilitation exercises.

 Also, we have added several elaborations in the results section, underpinning analytical findings regarding facilitators and barriers.

 Action taken:

 We have added Table 2 illustrating facilitators and barriers towards home-based rehabilitation exercises.

 We have elaborated on the results section to more clearly present findings regarding facilitators and barriers towards home-based rehabilitation exercises.

 No 6.

 Reviewer comments:

 Quote P12: “Because first of all…" – Could another interpretation be more like a desire for a contact to the healthcare system because of uncertainty?

 Reviewer comments:

 Thank you for suggesting an alternate interpretation of this quote. We believe it would be an overinterpretation to suggest that the participant refers to the healthcare system as a whole since this was said in relation specifically to the performance of the home-based rehabilitation exercises. Of course, we cannot rule out that there could be a connection to the healthcare system as well, but our data do not support this.

 Action taken:

 No action taken.

 No 7.

 Reviewer comments:

 To build upon what is already known, it could be interesting if there were any quotes about the last question in the interview guide: We are considering investigating whether this exercise program works better than general physical activities, what do you think of this idea?

 Author response:

 Thank you for your careful attention to the interview guide. The specific question was asked as a supplement with the purpose of gaining information regarding a possible future research study. Such a research study is currently being undertaken. Since the replies to the question do not provide answers to the aim of this study, no replies are included in this paper.

 Action taken:

 No action taken.

 No 8.

 Reviewer comments:

 The Discussion section is very short. Further discussion about the meaning of motivation, intrinsic and extrinsic factors could be valuable for the paper.

 Author response:

 Thank you for your suggestions regarding the discussion section, also including comments No. 9, 10, and 11. We agree that adding a perspective regarding intrinsic and extrinsic motivation could improve the discussion section, and we have added a passage concerning this.

 Action taken:

 The discussion section is extended and rewritten to a wide degree, including the perspectives of intrinsic and extrinsic motivation.

 No 9.

 Reviewer comments:

 The concept 'conduct of everyday life' was chosen as a theoretical framework, it would have been interesting to bring this framework into the discussion.

 Author response:

 Concerning this suggestion, we have elaborated on how returning to the well-known everyday life is used and described in relation to the concept of everyday life.

 Action taken:

 The discussion section is extended and rewritten to a wide degree, including the concepts of 'conduct of everyday life'.

 No 10.

 Reviewer comments:

 Additionally, topics such as pain and absence of pain and individual expectations alignment could be further discussed.

 Author response:

 Thank you for this suggestion. We have expanded the discussion regarding pain in the Discussion section.

 Action taken:

 The existing passage concerning pain has been extended, and now include the following:

*Some participants described a wish to have contact with a physiotherapist who would be able to provide guidance and support during the rehabilitation period. Positive impact of being in contact with a therapist during rehabilitation is supported in a review by Davenport et al. In terms of motivation this would serve as an extrinsic motivational factor for performing the home based rehabilitation exercises. Some of the participants in this study wished for contact to a physiotherapist, because they experienced barriers such as doubt of whether the exercises were performed correctly or whether to perform them at all e.g. due to pain. In such a situation the person might be fully motivated for performing the exercises on their own, but choose not to do so due to doubt regarding how to handle pain. According to Davenport et al, this would be accommodated when in contact with a therapist.*

*Specht et al showed that patients can have a feeling of uncertainty when being left alone to perform the rehabilitation exercise after discharge from the hospital, for example when dealing with pain. Our study adds the knowledge that not only pain, but also absence of pain can be identified as a barrier for performing home-based rehabilitation exercise. Standard care participants in this study gradually modified the exercises as they were able to return to their habitual everyday life, and performed the usual general physical activities they felt contributed to their quality of life instead. Modifying the exercises might also be a way of handling the fact that most participants described the exercises as boring. Modifying therapeutic instructions is well known in other areas, but to our knowledge, this is the first time it is described in THA patients.*

 No 11.

 Reviewer comments:

 To improve the Discussion section, more research and other theories on, for example, the above-mentioned topics could expand on the results and make implications for practice more clear.

 Author response:

 Thank you for the suggestion. We have addressed this by adding a section into the discussion, concerning perspectives on both implications for practice and for future research.

 Action taken:

 The following section is added to the discussion:

***Perspectives***

*Several perspectives grow out of our study both in terms of implications for practice and for further research.*

*Importantly, most participants appreciate the home-based rehabilitation exercise program underscoring the flexibility of deciding for themselves when and where to perform the exercises. Based on this, our study support the use of home-based rehabilitation exercise, but attention should be paid towards patients who might need additional support and easy access to a physiotherapist should be provided.*

*Concerning participants wish to return to their usual everyday lives and this serving as a motivational factor for performing the exercises, it is important that healthcare professionals are in dialogue with the individual patient to identify which activities are preferred in their habitual everyday life, and on that basis decide on relevant goals for the rehabilitation period.*

*Further, physiotherapists working in this area might want to consider how to include general physical exercise into rehabilitation exercise programs, since participants generally favoured this type of exercise over formal exercises. This might also address the finding of home-based rehabilitation exercises perceived as boring and thereby increase motivation and support adherence to the exercise program. Additionally, it would be useful to investigate whether general physical activities could be as effective as home-based rehabilitation exercise, and if so, whether future THA patients could rehabilitate by only doing their preferred physical activities.*

*There may be additional contributing factors in relation to patients’ perceptions of home-based rehabilitation exercise after THA. These could include age, gender, previous training experience, and culture. Further studies are needed to explore this.*

 No 12.

 Reviewer comments:

 Patients from the two groups were interviewed in different places (at the hospital and in their homes), what impact could that have on the results?

 Author response:

 Thank you for turning our attention to the potential impact of the setting of the interviews. We have added a passage addressing this point in the 'strengths and limitations' section.

 Action taken:

 The following passage is added to the section of strengths and limitations:

*The two groups of participants also differed regarding the location of the interviews. Participants recruited from usual care were interviewed in their homes, while participants recruited from PHETHAS-1 study were interviewed in a hospital setting. This might have affected data e.g. if participants were more comfortable in their homes as well as being in the setting where the home based rehabilitation exercises were performed and therefore would give more open hearted and detailed interviews.* *Data retrieved from interviews conducted in both types of settings are similar which indicate that this potential limitation is not applicable to this study.*

 No 13.

 Reviewer comments:

 In addition, patients being a part of another study, what consequences does that have for the transferability of the study?

 Author response:

 We have been transparent throughout the paper about recruiting from two groups of participants. Importantly, the only difference was some participants being part of another study. This recruitment strategy had a positive impact, and we have accounted for potential negative impacts. Meanwhile, we have elaborated on these points in the discussion section.

 We do view results as transferable to similar settings, although one could argue for a higher degree of transferability regarding findings from participants recruited from usual care. Yet, the only difference in our findings was a higher degree of motivation towards adherence over time and performing exercises as instructed, which is accounted for in the discussion section.

 Action taken:

 We have added the following passage in italics to the strengths and limitations section:

*In this study we recruited participants from both the PHETHAS-1 study and from standard care. This combination of participants revealed an important difference in motivation towards adherence to, and performance of, the home-based rehabilitation program. The PHETHAS-1 participants had* *a higher degree of motivation towards adherence over time and performing exercises as instructed, but* *our findings showed no other differences between the two groups of participants. In addition to this specific point, one might argue that findings from the PHETHAS-1participants have a lower degree of transferability to practice compared to the usual care participants.*

 No 14.

 Reviewer comments:

 Two paragraphs in the Discussion section belong more to the Conclusion or a perspective section: “Overall the participants …” and “There may be additional …”.

 Author response:

 Thank you for this fine suggestion.

 Action taken:

 The two paragraphs are moved to the new 'perspectives' section.

 No 15.

 Reviewer comments:

 The conclusion is supported by the results. However, adding a perspective section would be helpful to clarify how the findings can improve clinical practice.

 Author response:

 Again, thank you for this suggestion which we have met by adding a 'perspectives' section.

 Action taken:

 A 'perspectives' section is added.
